# Efficacy and safety of combined loratadine and mometasone furoate therapy in allergic rhinitis patients

**DOI:** 10.3389/fimmu.2025.1560295

**Published:** 2025-06-04

**Authors:** Hui Yong, Lingling Di, Zhikai Wang, Jing Yang, Pei Yang, Xiaoping Gao

**Affiliations:** Department of Otorhinolaryngology Head and Neck Surgery, General Hospital of Ningxia Medical University, Yinchuan, China

**Keywords:** loratadine, mometasone furoate, combination therapy, allergic rhinitis, efficacy

## Abstract

**Objective:**

This study seeks to assess the effectiveness and safety of a combination treatment involving loratadine and mometasone furoate for patients suffering from allergic rhinitis (AR). Additionally, it explores the risk factors contributing to treatment failure, providing a theoretical basis for identifying safer and more effective AR treatments.

**Methods:**

A prospective study was carried out between January 1, 2021, and April 1, 2023, involving 116 patients with allergic rhinitis (AR) who were treated at our outpatient clinic. Participants were randomly divided into two groups: the control group (n=58), which received loratadine alone, and the study group (n=58), which received a combination of loratadine and mometasone furoate. Outcome measures included nasal symptom scores and serological markers, assessed before and after the treatment period. The effectiveness of the treatment was assessed using nasal symptom scores.

**Results:**

Post-treatment assessments showed that both nasal symptom scores and serological markers were significantly lower in the study group compared to the control group (P<0.05). Additionally, the overall response rate was markedly higher in the study group (P<0.05). There were no significant differences in the total incidence of adverse reactions between the two groups (P>0.05).

**Conclusion:**

The combination of loratadine and mometasone furoate effectively alleviates clinical symptoms in patients with allergic rhinitis while demonstrating a favorable safety profile, making it a promising option for clinical use.

## Introduction

Allergic rhinitis (AR) is a prevalent condition encountered in clinical settings, characterized primarily as a non-infectious inflammatory disorder of the nasal mucosa that is mediated by elevated levels of immunoglobulin E (IgE) in response to allergens (1). Approximately 500 million individuals globally are affected by AR, and this number continues to rise annually (2). The traditional pathophysiology of AR involves allergic responses triggered by an imbalance between Th1 and Th2 immune cells (1). The clinical manifestations of mild AR include sudden sneezing, nasal itching, nasal congestion, runny nose, etc., and continuous progression of the disease can develop into complications such as bronchial asthma, chronic sinusitis, and secretory otitis media (1). Patients with severe AR may present with symptoms such as severe dizziness, headache, and memory loss (1). Though AR does not endanger the patient ‘s life, it can seriously affect patients’ quality of life and mental health.

With a wide variety of them, nasal glucocorticoids are currently the most effective drugs for the treatment of AR, and guidelines have not clearly recommended the specific selection (3, 4). Mometasone furoate is a nasal glucocorticoid used more frequently in clinical practice, which has the effects of anti-allergy, inhibiting cell division, and anti-inflammation. It enters the human body and binds to hormone receptors in the cytoplasm, which can rapidly improve the clinical symptoms of patients, smooth the nasal passage, is non-irritating, and has a high patient acceptance (5). Loratadine, an H1 receptor blocker, is a long-acting tricyclic antihistamine that specifically selects peripheral H1 receptors and competitively inhibits a variety of allergic reactions caused by histamine. After entering the body, it can inhibit P-1 selectin, regulate serum soluble intercellular adhesion molecule-1, reduce intercellular adhesion molecule expression, and weaken inflammatory cell activation and release (6). Previous studies have determined that loratadine in combination with other therapies can effectively relieve the clinical symptoms of AR patients (7). Yao believed that allergen blocking agent combined with mometasone furoate nasal spray in the treatment of AR can effectively improve the nasal physiological function (8). Currently, there were few clinical studies on loratadine combined with mometasone furoate in the treatment of AR patients, especially fewer studies on prognosis.

In light of the significant burden that allergic rhinitis (AR) places on patient quality of life and the limitations of current treatments, this study aims to rigorously assess the efficacy and safety profile of combining loratadine with mometasone furoate. By identifying key risk factors for treatment failure, our findings aspire not only to enhance clinical outcomes but also to pave the way for future therapeutic innovations in AR management. This study endeavors to contribute valuable insights towards the ultimate goal of developing more personalized and effective treatment strategies for AR.

## Materials and methods

### Participants

From January 1, 2021, to April 1, 2023, this prospective study included 116 patients with allergic rhinitis (AR) who were treated at our outpatient clinic. Using a random number table, participants were assigned to either the control group (n = 58) or the study group (n = 58). Those in the control group received loratadine, while the study group was treated with a combination of loratadine and mometasone furoate. This design was implemented to evaluate the incremental efficacy of adding mometasone furoate in mild-moderate persistent AR patients, where antihistamine monotherapy remains an accepted option per current guidelines (9). The study protocol was developed in accordance with the principles outlined in the Declaration of Helsinki by the World Medical Association. It received approval from the Ethics Committee of the General Hospital of Ningxia Medical University (Approval Number: 2020R1208), and informed consent was obtained from all participants.

### Inclusion and exclusion criteria

#### Inclusion criteria

1. Patients who fulfill the diagnostic criteria for allergic rhinitis (AR) as outlined in the Chinese Guidelines for the Diagnosis and Treatment of Allergic Rhinitis (2022, revised edition) (9).
2. Patients who have not received treatment with glucocorticoids or antihistamines prior to enrollment.
3. Patients with no known allergies to loratadine or mometasone furoate.
4. Patients who have provided informed consent.

#### Exclusion criteria

1. Patients who did not complete the treatment course.
2. Patients with asthma, bronchial issues, or other respiratory diseases.
3. Pregnant or breastfeeding women.
4. Patients diagnosed with other malignant tumors or infectious diseases.
5. Patients with severe organic diseases affecting the heart, liver, or kidneys.
6. Patients with immune deficiencies.
7. Patients with nasal polyps, sinusitis, or any other abnormal nasal anatomy.

Participants were fully informed about study procedures and provided written consent before enrollment. Active involvement included:

- Weekly clinical assessments during the 4-week treatment period
- Daily symptom diaries documenting nasal congestion, rhinorrhea, and sneezing
- Medication adherence verification through returned blister pack counts
- Optional post-study follow-up for outcome persistence evaluation

### Treatment protocol

All patients were given nasal irrigation with normal saline (Nasalcare, registration certificate No.: Su Xie Zhu Zhun 2016214000). Both nasal cavities were irrigated daily in the morning with 240 mL each time.

The control group received loratadine monotherapy (10 mg/day) as this regimen is recommended for mild-moderate cases in both Chinese and ARIA guidelines when nasal steroid intolerance exists or for patients with predominant histamine-mediated symptoms (9). Loratadine (Hainan Haishen Tongzhou Pharmaceutical Co., Ltd., 2006281), orally, once daily, 10 mg each time, was administered 1 hour before bedtime. The treatment was continued for 1 month.

The study group was treated with loratadine combined with mometasone furoate. Mometasone furoate (Schering-Plough Labo N.0214000H1) was administered once daily at bedtime as 1 spray (100 μg unilaterally) into each nostril. If the clinical symptoms of patients were relieved, the dose could be reduced to 1 spray (50 μg unilaterally) in each nostril. The above treatment was continued for 1 month.

### Outcome indicators

Basic clinical characteristics were gathered for all participants. The primary outcome measures included nasal symptom scores and serological markers, which were assessed both before and after the treatment course. The effectiveness of the treatment was determined based on the nasal symptom score.

#### Nasal symptom score

The nasal symptoms of obstruction, itching, sneezing, and runny nose were evaluated according to the Chinese Guidelines for the Diagnosis and Treatment of Allergic Rhinitis (2022, revised edition) (9). The scoring system is as follows:

- **Nasal Obstruction Score:**
- 1 point: occasionally
- 2 points: frequently
- 3 points: nearly mouth breathing
- **Nasal Itching Score:**
- 1 point: intermittent
- 2 points: tolerable
- 3 points: intolerable
- **Sneezing Score:**
- 1 point: 3–9 times
- 2 points: 10–14 times
- 3 points: 15 times or more
- **Runny Nose Score:** Based on the daily frequency of wiping:
- 1 point: ≤ 4 times
- 2 points: 5–9 times
- 3 points: ≥ 10 times

#### Serological indicators

The following markers were evaluated: IgE, interleukin (IL)-6, IL-10, and transforming growth factor-β1 (TGF-β1). Measurements were taken at baseline (pre-treatment) and post-treatment (after 1 month). A venous blood sample of 5 ml was obtained from each patient. The enzyme-linked immunosorbent assay (ELISA) technique was employed, with kits sourced from Shanghai Enzyme-linked Biotechnology Co., Ltd. All procedures were conducted in strict accordance with the provided instructions.

#### Treatment outcomes

Treatment effectiveness was assessed based on the nasal symptom score:

- Significantly Effective: > 80% reduction in total nasal symptom score
- Effective: 50% - 79% reduction in total nasal symptom score
- Ineffective:< 50% reduction in total nasal symptom score

The overall response rate was calculated as follows: (significantly effective cases + effective cases)/total cases × 100%.

#### Adverse effects

Data regarding drug-related adverse reactions were collected from all patients, with the main reported effects including sore throat, drowsiness, palpitations, and gastrointestinal irritation.

### Statistical analysis

Data collected during this study were analyzed using SPSS version 26.0. The normality of continuous variables was assessed using the Shapiro-Wilk test, along with graphical representations such as histograms and Q-Q plots. Measurement data that followed a normal distribution were reported as mean ± standard deviation (SD), while non-normally distributed data were presented as median (interquartile range). Comparisons between groups were conducted using the Student’s t-test for normally distributed data and the Mann-Whitney U test for non-parametric data. Categorical data were expressed as n (%), and differences between the two groups were analyzed using chi-square tests or Fisher’

Exact Test. A significance level of 0.05 was established for two-sided tests. Sample size calculations were performed using the formula


$$n=\frac{2\sigma^{2}{\left(Z_{1-\alpha /2}+Z_{1-\beta }\right)}^{2}}{s^{2}}+(1+dropout\;\;rate)$$

where σ = pooled standard deviation (derived from pilot data), s = clinically meaningful difference (effect size), α = 0.05 (Type I error), β = 0.01 (Type II error; power = 99%), and a 15% anticipated dropout rate. This yielded a minimum required sample of 122 participants.

## Results

### Baseline clinical characteristics

A total of 116 patients with allergic rhinitis (AR) were included in the study. The study group comprised 58 patients aged between 22 and 68 years, with a mean age of 47.09 ± 15.23 years; this group included 37 males and 21 females. The control group also consisted of 58 patients, aged 23 to 69 years, with a mean age of 47.16 ± 15.99 years, including 35 males and 23 females. There were no significant differences in other baseline clinical characteristics between the two groups (P > 0.05). (See **Table 1**).

**Table 1:** Baseline clinical characteristics.

Clinical characteristics	Study group (n=58)	Control group (n=58)	χ^2^/t	*p*
Males	37 (63.79%)	35 (60.34%)	0.146	0.702
Age (years)	47.09±15.23	47.16±15.99	-0.024	0.981
Smoking	12 (20.70%)	9 (15.52%)	0.523	0.469
Drinking	7 (12.07%)	10 (17.24%)	0.621	0.421
Tend to favor cereals, starch, and vegetables	37 (63.79%)	31 (53.45%)	1.279	0.258
Pollen allergy	26 (44.83%)	23 (39.66%)	0.318	0.573
HDM allergy	22 (37.93%)	18 (31.03%)	0.611	0.435
Air bedding frequently	27 (46.55%)	30 (51.72%)	0.309	0.577
Sleep more than 8 hours/day	32 (55.17%)	29 (50.00%)	0.311	0.577
Keep pets	23 (39.66%)	27 (46.55%)	0.562	0.453
Open Windows frequently for ventilation	42 (72.41%)	38 (65.52%)	0.644	0.422
History of food allergy	39 (67.24%)	34 (58.62%)	0.924	0.336
Damp living environment	32 (55.17%)	36 (62.07%)	0.569	0.451
History of asthma	28 (48.28%)	33 (56.90%)	0.864	0.353
Indoor flower growing	32 (55.17%)	29 (50.00%)	0.311	0.577
Nasal symptom score before treatment (points)				
Nasal obstruction	7.32±0.67	7.29±0.63	0.248	0.805
Nasal itching	7.67±0.73	7.61±0.71	0.449	0.654
Sneezing	7.83±0.59	7.76±0.63	0.618	0.538
Runny nose	7.79±0.65	7.68±0.56	0.976	0.331
Serologic indicators before treatment				
IgE (U/ml)	421.29±21.29	419.87±23.18	0.344	0.731
IL-6 (ng/ml)	10.37±1.24	10.31±1.23	0.262	0.794
IL-10 (ng/ml)	29.38±2.01	29.32±1.97	0.162	0.872
TGF-β1 (ng/L)	429.52±38.91	427.38±37.67	0.301	0.764

HDM, House Dust Mite; IL, Interleukin; TGF, Transforming growth factor.

### Comparison of nasal symptom scores after treatment

Post-treatment, the nasal symptom scores for nasal obstruction, itching, sneezing, and runny nose in the study group were significantly lower than those in the control group (P< 0.05). (Refer to **Table 2**).

**Table 2:** Comparison of nasal symptom score after treatment.

Nasal symptom score (points)	Study group (n=58)	Control group (n=58)	χ^2^/t	*p*
Nasal obstruction	1.46±0.43	2.32±0.39	-11.282	<0.001
Nasal itching	1.32±0.23	2.34±0.27	-21.901	<0.001
Sneezing	1.27±0.38	2.48±0.41	-16.484	<0.001
Runny nose	1.09±0.32	2.54±0.43	-20.602	<0.001

### Comparison of serological indicators after treatment

Following treatment, the levels of IgE, IL-6, IL-10, and TGF-β1 in the study group were significantly reduced compared to those in the control group (P< 0.05). (See **Table 3**).

**Table 3:** Comparison of serologic indicators after treatment.

Serologic indicators	Study group (n=58)	Control group (n=58)	χ^2^/t	*p*
IgE (U/ml)	117.29±23.34	157.43±22.26	-9.478	<0.001
IL-6 (ng/ml)	5.18±1.28	7.38±1.32	-9.112	<0.001
IL-10 (ng/ml)	14.28±1.87	12.18±1.79	6.178	<0.001
TGF-β1 (ng/L)	271.29±34.29	323.87±35.39	-8.126	<0.001

IL, Interleukin; TGF, Transforming growth factor.

### Comparison of treatment outcomes

The overall response rate was significantly higher in the study group than in the control group (P< 0.05). (Refer to **Table 4**).

**Table 4:** Comparison of treatment outcomes.

Treatment outcomes	Study group (n=58)	Control group (n=58)	χ^2^/t	*p*
Significantly effective	31 (53.45%)	22 (37.93%)		
Effective	26 (44.83%)	29 (50.00%)		
Ineffective	1 (1.72%)	7 (12.07%)		
Overall response	57 (98.28%)	51 (87.93%)	4.833	0.028

### Comparison of adverse effects

**Table 5** summarizes the drug-related adverse reactions observed in both groups. The overall incidence of adverse reactions did not differ significantly between the two groups (P > 0.05).

**Table 5:** Comparison of adverse effects.

Drug-related adverse reactions	Study group (n=58)	Control group (n=58)	χ^2^/t	*p*
Sore throat	1 (1.72%)	3 (5.17%)		
Drowsiness	1 (1.72%)	2 (3.45%)		
Palpitations	0	1 (1.72%)		
Gastrointestinal irritation	3 (5.17%)	4 (6.90%)		
Total	5 (8.62%)	10 (17.24%)	1.914	0.166

## Discussion

AR arises from nasal mucosal inflammation mediated predominantly by IgE antibodies (10). Initial allergen exposure triggers IgE production, which binds to eosinophils and mast cells, inducing sensitization (10). Upon re-exposure, these cells release bioactive mediators (e.g., interleukins, histamine), leading to vascular permeability, smooth muscle spasm, and glandular hypersecretion, ultimately impairing respiratory and auditory function (11). Uncontrolled AR may progress to complications such as asthma, conjunctivitis, and vascular endothelial injury, severely impacting quality of life. Current treatment strategies focus on modulating allergic responses or enhancing tolerance to allergens (12), though seasonal variability complicates long-term management (12). While immunotherapy is effective for patients with defined immunogens (13), pharmacotherapy remains central for those with unidentified triggers. Common agents include glucocorticoids, antileukotrienes, and antihistamines (14).

Mometasone furoate, a potent topical glucocorticoid, exerts anti-allergic effects by reducing capillary permeability, inhibiting inflammatory cell migration, and suppressing cytokine release (15). Its low bioavailability minimizes systemic adverse effects, making it suitable for chronic use (15). Studies demonstrate rapid onset (7 hours) and sustained efficacy (16), with Wang et al. confirming its superiority over monotherapy in alleviating AR symptoms (17). Loratadine, a second-generation antihistamine, antagonizes peripheral H1 receptors, mitigating leukotriene-mediated inflammation (18). However, its efficacy diminishes in severe AR due to limited action on eosinophil activity (19), necessitating combination therapies (20). In this study, loratadine combined with mometasone furoate achieved a 98.28% overall response rate (53.45% significantly effective + 44.83% effective; vs. 87.93% for loratadine alone; P = 0.028; NNT = 9), aligning with Dai et al.’s findings (21).

Zhang et al. (22) emphasized that loratadine combinations enhance symptom control in AR. Our results corroborate this: nasal obstruction scores decreased from 7.32 ± 0.67 to 1.46 ± 0.43 points (80.1% reduction), itching from 7.67 ± 0.73 to 1.32 ± 0.23 (82.8%), sneezing from 7.83 ± 0.59 to 1.27 ± 0.38 (83.8%), and runny nose from 7.79 ± 0.65 to 1.09 ± 0.32 (86.0%) in the study group (all P< 0.001), exceeding the MCID threshold of 0.5 points (23). These reductions reflect meaningful clinical improvement, as symptom severity directly correlates with quality-of-life metrics (24).

Serologic analyses revealed significant reductions in IgE (117.29 vs. 157.43 U/mL; *P*< 0.001), IL-6 (5.18 vs. 7.38 ng/mL; *P*< 0.001), and TGF-β1 (271.29 vs. 323.87 ng/L; *P*< 0.001). Notably, IL-10 levels were paradoxically higher in the study group (14.28 vs. 12.18 ng/mL; *P*< 0.001), contrasting with Wan et al.’s report of IL-10 suppression (25). Notably, IL-10 levels in the study group decreased from 29.38 ± 2.01 to 14.28 ± 1.87 ng/mL post-treatment, a less pronounced reduction compared to the control group (29.32 ± 1.97 to 12.18 ± 1.79 ng/mL; P< 0.001). This paradoxical relative elevation may reflect mometasone’s dual anti-inflammatory modulation (26). Similarly, Xie et al. observed TGF-β1 modulation with similar combinations (27), supporting our findings.

The significant reduction in both nasal symptom scores and serological markers in the study group suggests that the combination of loratadine and mometasone furoate is not only effective in alleviating clinical symptoms but also beneficial in reducing systemic inflammation, thereby addressing one of the primary aims of this study: to evaluate the efficacy of combined therapy in managing allergic rhinitis. Furthermore, our finding that the overall response rate was markedly higher in the study group (P = 0.028) underscores the potential of this combined treatment strategy as a promising option for AR management, aligning closely with our goal to identify safer and more effective therapeutic options for patients suffering from allergic rhinitis.

Safety profiles were comparable between groups, with low rates of sore throat (1.72% vs. 5.17%), drowsiness (1.72% vs. 3.45%), and gastrointestinal irritation (5.17% vs. 6.90%; total 8.62% vs. 17.24%; P = 0.166), consistent with Da et al.’s conclusion (28).

This study has several limitations. One limitation of this study is that the small sample size may limit the generalizability of the findings. Additionally, the shorter follow-up duration restricts the ability to detect potential long-term differences in combination therapy. Future research should incorporate a larger, randomized, and blinded study design to further investigate the possible relationships in allergic rhinitis patients receiving combination therapy. Finally, while nasal irrigation represents an evidence-based adjunct therapy for certain AR presentations, our study intentionally excluded irrigation to isolate the comparative effects of systemic versus topical pharmacotherapy. Future studies could evaluate whether adding saline irrigation provides incremental benefit to these medication regimens.

## Conclusion

In summary, loratadine combined with intranasal mometasone furoate achieved marked symptom relief (nasal obstruction: 7.32→1.46 points; runny nose: 7.79→1.09 points) and reduced inflammatory biomarkers (IgE: 421.29→117.29 U/mL; IL-6: 10.37→5.18 ng/mL) in AR patients, with a favorable safety profile (NNT = 9; adverse reaction rate = 8.62%). These findings support the superiority of combined therapy over loratadine monotherapy (87.93% response rate) and its clinical adoption for moderate-to-severe AR.

## Data Availability

The original contributions presented in the study are included in the article/supplementary material. Further inquiries can be directed to the corresponding authors.
